# Molecular assessment of food web dynamics identifies critical periods for managing resilience in biological pest control

**DOI:** 10.1002/eap.70078

**Published:** 2025-07-31

**Authors:** Pedro Nuno Branco Leote, Oskar Ragnar Rennstam Rubbmark, Michael Traugott

**Affiliations:** ^1^ Applied Animal Ecology Research Unit, Department of Zoology University of Innsbruck Innsbruck Austria

**Keywords:** behavior‐constrained, behavior‐free, biological control, molecular gut content analysis, specialization, time series food webs

## Abstract

Food webs are not static over time, but our knowledge of their dynamics is extremely scarce due to methodological challenges. In turn, this significantly limits our ability to mechanistically understand the temporal changes that trophic networks annually undergo. Here, we address this gap using DNA‐based diet analysis to measure the season‐wide dynamics of trophic interactions between invertebrate generalist predators, pest, and alternative prey in replicated cereal fields across 2 years. We used the level of food web specialization as a proxy for predator diet overlap in pest control and hypothesized that it would reach its minimum at the middle point of the season, when primary production should be higher (H1). Conversely, invertebrate diversity would reach its maximum during the same period (H2). Additionally, alternative prey availability would be indirectly increased by adding manure to half of each field to test if this would reduce specialization and increase diversity (H3). In line with our predictions, food web specialization was lowest during the middle of the season, when prey, but not predator, diversity reached its maximum. No significant effects of manure addition were found on food web specialization. Our findings suggest early and late season in cereal systems as the times when generalist predators are behaviorally most constrained, pinpointing these as periods when the pests are eaten by a smaller subset of the predator community. Hence, molecular trophic analyses provide unique insights into the temporal dynamics of food webs and their properties. This allows the generation of temporal roadmaps for when management interventions are expected to be most effective.

## INTRODUCTION

Biological communities are defined by the changes that occur over time and space in response to environmental conditions, resources, and interactions between species. Many of those interactions are antagonistic and often of a predator–prey nature (Schmitz, 2017). These interactions occur at an individual level and are at least partially responsible for changes in population geographic range, abundance, community structure, evolution, and behavior (Kishida et al., 2010; Schmitz et al., 1997, 2004). Even though population regulation responses are often studied from the perspective of mortality and recruitment from reproduction that occur over time, this is not necessarily what occurs on short time scales (e.g., within the same year or season). In such cases, recruitment can occur in the form of migration or behavioral adaptation. The latter can take place when changes in the behavior of multiple species cause an increase in their diet overlap and, in turn, increase the number of species consuming a given food source. Both migration and behavior would result in a greater number of individuals and species diversity involved in the trophic interactions (Birkhofer et al., 2015).

Moreover, when induced across spatial and temporal scales, predator–prey interactions create a constant feedback loop (Dingemanse & Wolf, 2013; Webster, 2003), that can be split into periods when predators' behavior is free or constrained (Barbier & Loreau, 2019). Behaviourally constrained periods are akin to bottom‐up regulation, but from the perspective of the forces that drive behavior, as opposed to those that shape community structure. More specifically, bottom‐up and behaviourally constrained periods occur when the resources available are limiting, and restrict consumer populations on the successive trophic levels (Hunter & Price, 1992, Power, 1992). Behaviorally, this limitation can be because resources, such as food sources, are not present in sufficient amounts, leading to higher intra‐ and interspecies competition that limits predators' access to those resources (Oksanen, 1988). Interspecies competition can force species to adapt their niches to avoid competitors by changing their behavior, causing species to reduce their dietary overlap, effectively increasing the network specialization within a food web (H2′—network‐level specialization in Blüthgen et al., 2006). Conversely, behaviorally free periods would occur when there is plenty of a resource, which in turn releases consumers and predators from competition. This enables consumers to overlap their diets more, and reduce food web specialization. This would increase the functional redundancy of top‐down regulation, by increasing the number of predator species targeting prey (Biggs et al., 2020; Lawton & Brown, 1993; Loreau, 2004). If prey are pests, such top‐down regulation becomes a form of biological control, that can be relevant from an economic perspective.

However, the empirical study of behaviorally free and constrained periods in real‐world food webs requires a great deal of sampling effort in order to represent the systems accurately (De Barba et al., 2014), especially when dealing with generalists. Such high temporal resolution in food webs requires a standardized, large‐scale assessment of many feeding links between multiple prey and predator species, an endeavor challenged by the difficulties inherent to identifying prey remains at high specificity and sensitivity (Nielsen et al., 2018). Molecular methods have revolutionized the study of trophic interactions (Symondson, 2002), as they allow examining trophic links with great accuracy, many of which are intractable by other methodologies. More importantly, they allow us to scale up a standardized analysis of diet samples to measure food webs with thousands of individual consumers (Deagle et al., 2023). In fact, we are now at a point when it is not so much the ability to describe food webs, but rather how much temporal resolution the data should have in order to answer specific questions on food web variability, given the discrete nature of sampling (Pringle & Hutchinson, 2020).

Therefore, a lack of understanding of the temporal dynamics of food webs has been pointed out (Isbell et al., 2018; Iuliano & Gratton, 2020), due to past research focusing on limited time windows (McMeans et al., 2015; Roubinet et al., 2018), and of empirical studies on how biological control is built up and regulated throughout an entire growing season (Barbier & Loreau, 2019; Cohen & Crowder, 2017; Roubinet et al., 2018; Staudacher et al., 2018; Welch & Harwood, 2014). Both relate to the temporal resolution of data, allowing the detection of changes over time in food webs. In the case of biological control, this information may guide management interventions, such as when and how to fertilize or till the soil, to boost natural enemy populations and create food webs that maximize trophic pressure on pests.

For that reason, food web temporal dynamics are especially relevant for conservation biological control (CBC), as we still lack a solid understanding of which mechanisms drive its efficacy and robustness. Being linked to natural enemy diversity (Crowder et al., 2010), the more species contributing to CBC (thus, higher functional redundancy), the higher the likelihood that this service is retained if one species disappears (insurance hypothesis, Yachi & Loreau, 1999). The other half of the equation is the prey. Increasing their overall abundance should reduce predator competition, which in turn would reduce constraints on predator behavior and dietary niches and decrease specialization.

In order to assess how and when this occurs under natural conditions, we conducted a study to record the food web dynamics, and behaviorally constrained and free periods in barley (*Hordeum vulgare* L.) fields. We did this by sampling every 2 weeks across the growing season of the cereal during two consecutive years. We examined invertebrate food webs, with generalist predators (beetles and spiders), non‐pest (earthworms and springtails) and pest prey (three species of aphids and a cereal leaf beetle), which are ubiquitous in cereal fields in Central Europe (Bokova et al., 2023; Dedov et al., 2006; Dinter et al., 2013; Mancini et al., 2023; Vickerman & Wratten, 1979; Wolters, 2001). Each field had half of its area fertilized with manure to create a difference in baseline primary production. Furthermore, the manure treatment was selected to increase mainly the abundance of non‐pest detritivore prey and induce a behavior release for predators (Rowen et al., 2019), thereby reducing their specialization. The use of high‐throughput multiplex polymerase chain reaction (PCR) assays for molecular gut content analysis (MGCA) allowed us to examine the diets of the several thousand generalist predators and generate a unique time series of empirically established and replicated trophic networks to see how food web specialization fluctuates over time.

In a process similar to ecological succession, the fields develop from nearly empty, with just seedlings, to a green, tall, homogenous cover, before drying out later on. As the season progresses, and primary production and plant biomass increase, so too should prey abundance. This was reinforced in our system with the addition of cattle manure to boost plant growth (Miller et al., 2009), as well as decomposers and detritivores (Aguilera et al., 2021; Riggi & Bommarco, 2019), which have been shown to decrease pest abundance (Arancon et al., 2007; Rowen et al., 2019; Yardım & Edwards, 2003). We have also previously shown this increase in non‐pest prey catches and pest suppression due to the application of manure in a previous paper focusing on competition, intraguild predation, and CBC, using the same dataset (Leote et al., 2024). Furthermore, manure should create a more diverse prey community to attract more predators to fields, and in turn increase the latter's diversity as well (Winqvist et al., 2011). However, given the short‐term duration of the sampling in this study, we expect this effect to be relatively weak when compared to longer term studies, as numerical responses from predators due to increased reproduction would not come into play. Nonetheless, with a greater predator diversity and dietary overlap (lower specialization), the likelihood of multiple species exerting control over pest prey increases. As such, to expand upon our previous results (Leote et al., 2024), in this article we focus on the network specialization, predator and prey diversity, with the purpose of identifying periods of greater vulnerability for CBC.

**FIGURE 1:**
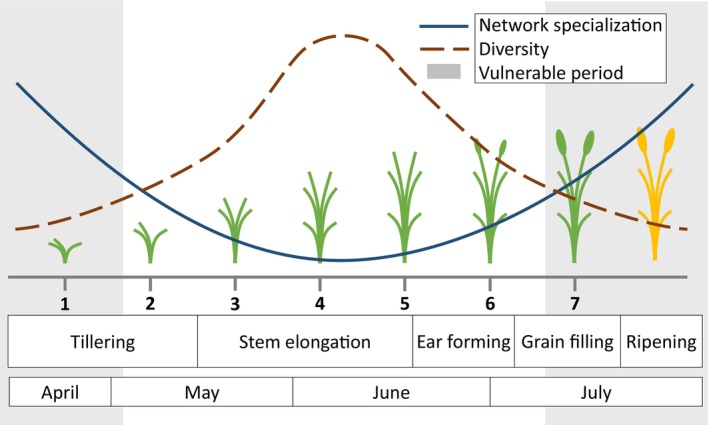
Predictions of network specialization (full blue line), invertebrate community diversity (dashed brown line) and vulnerable periods for biological control (shaded areas), throughout the season (in months) and sampling sessions, or time points (in numbers), in relation to barley growth stages. Illustration credit: Pedro Nuno Branco Leote.

Using this setup, and taking the functional link between diversity and CBC (Begg et al., 2017), we have derived three hypotheses for our study. Our first hypothesis is regarding the food webs' network specialization: when generalist predators establish themselves in the nearly empty field, during early cereal tillering, their behavior should be relatively specialized. This is likely due to the low prey abundance and resulting competition (Leote et al., 2024) that constrain predator behavior. Afterward, as the cereal grows, the habitat becomes more vertical and structurally complex, and prey abundances increase (Nagel, 1979). Consequently, predators should be freer to adapt their behavior, expand their dietary niches, and become more generalized (Snyder et al., 2022). Later, when the cereal dries and prey decrease, predators should become more constrained and specialized again (H1, Figure 1).

Our second hypothesis is analogous to the first, but focuses on the diversity of invertebrates, rather than specialization. As the season progresses, and primary production and plant biomass increase, so too should prey abundance and diversity (Douglas et al., 2010; Leote et al., 2024; Smith et al., 1985), and should bring more predators to fields. Therefore, we expect predator and prey diversity to increase at the start, reaching its maximum during mid‐season, then decrease toward the end (H2, Figure 1).

In our third hypothesis, we predict food web specialization should be lowest, and invertebrate diversity highest, with the manure treatment (H3). As stated above, the increase in primary production that is expected to occur over the season should lead to higher prey abundance and diversity, and in turn allow a higher predator diversity and lower specialization. Thus, our rationale for this hypothesis is simple: a treatment with a higher baseline primary production should display a more pronounced effect on predator and prey diversity and predator specialization. Through these hypotheses, we aim to address the knowledge gaps on the temporal dynamics of regulation phases, the diet overlap of generalist predators and their behavioral constraints, as well as the build‐up of biological control over time.

## MATERIALS AND METHODS

### Field sampling

**FIGURE 2:**
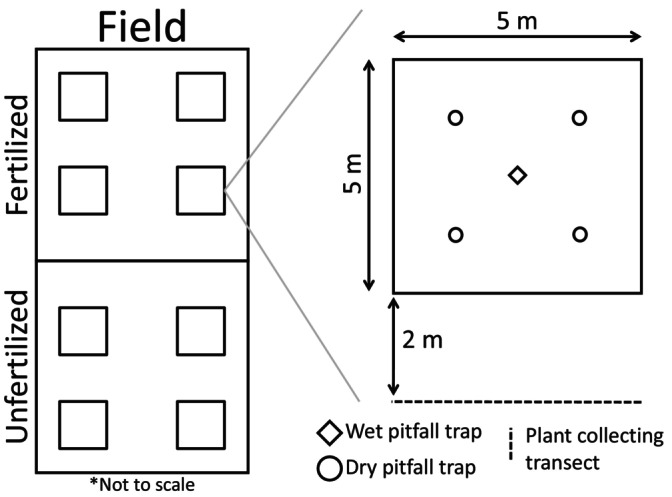
Schematic of sampling plot distribution within fields (not to scale), and sampling methods within plots. One half of each field was fertilized with manure at a rate of 1.5 t/ha, and the other half was left untreated. Wet and dry pitfall traps were used to assess the community and predator diet, respectively, while plant collecting transects were done to determine the number of aphids present in the fields. Sampling plots were at least 5 m from the field edge, so as to avoid potential edge effects of the field. They were also at least 10 m away from one another along the field's width (horizontal axis in this diagram), between 15 and 20 m along the length of the field (vertical axis) depending on its total length. Lastly, plots were set at least 25 m away from the border between the two treatments within the same field.

The study was conducted in Kematen in Tirol, Austria, where spring barley (*H. vulgare* L.) was grown in six fields that have had organic management for the past 10 years, three in 2020 and 2021, respectively. Each field was split in half, one side being fertilized with manure and the other remaining unfertilized, as a negative control. The manure treatment was independently applied by each field's respective owner, at a standard rate of 1.5 metric tons (1500 kg) per hectare, using manure spreaders, after tilling and pressing the soil. Four 5 × 5 m plots per treatment were delimited to carry out all sampling (Figure 2). These plots had no barriers or fences surrounding them that would impede or limit movement between the inside of the plots and the rest of the field area. Additionally, the plots were at least 5 m from the field edge to avoid edge effects. They were at least 10 m away from each other along the field's width and between 15 and 20 m along the length of the field. To avoid any spillover of treatment effects, plots were set at least 25 m away from the border between the two treatments within the same field.

Sampling was conducted every 2 weeks, with 1 week of sampling and one of rest. The sampling period started on 21 April and ended on 14 July in 2020, while it started on 3 May and ended on 12 July in 2021, to encompass almost the entire crop season. This resulted in seven sampling time points for the first year and six for the second, which we refer to as sessions throughout this article and in the figure captions. Dry pitfall traps with wood chips were set up and remained active for a single day to catch live predators to conduct the molecular gut content analysis (Staudacher et al., 2016). The *taxa* collected were ground beetles (Carabidae), rove beetles (Staphylinidae) and spiders (Araneae). Wet pitfall traps, with a teaspoon of salt (5–6 g) and a drop of detergent (approx. 1 mL) per liter of water, remained active for 4 days to sample the soil surface dwelling community (see Appendix S1: Tables S1–S3 for list of *taxa* obtained from pitfall traps). A total of 168 wet pitfall trap samples were obtained in 2020 (4 sampling plots × 2 treatments × 3 fields × 7 sampling sessions) and 144 for 2021 (4 × 2 × 3 × 6 sampling sessions), from which Shannon‐Weaver's diversity was calculated for the predator and prey communities. Additionally, transects were carried out along the outside border of the plots (5 m) to avoid trampling on the inside, collecting 30 individual barley plant tillers to count the number of aphids per tiller. Since aphids are mostly plant‐dwelling, unlike the majority of the other *taxa* sampled, these tiller counts replaced the values obtained for aphids from the wet pitfall traps in the diversity calculations described further down below in *Invertebrate diversity*, as they better represented their abundances.

### Molecular gut content analysis

A total of 2404 ground beetles, 913 rove beetles, and 567 spiders were captured in 2020, and 1977 ground beetles, 891 rove beetles, and 250 spiders in 2021. The beetles' gut content and the spiders' full bodies were extracted with a BioSprint 96 DNA Blood Kit (Qiagen, Hilden, Germany) on a QIAGEN Biosprint96 workstation for automated DNA extraction, following the manufacturer's recommendations. After extraction, three different multiplex PCR assays were run per sample. The first assay targeted several prey *taxa* (assay in Rennstam Rubbmark et al., 2019), the second targeted generalist and specialist predators, such as spiders and ladybeetles, as intra‐ and extraguild prey (primers from Sint et al., 2014; Staudacher et al., 2016). Lastly, the third assay identified the genus of beetles consumed, also as intraguild prey, from a selected set of common *taxa* consisting of *Bembidion* spp., *Harpalus* spp., *Poecilus* spp., *Pterostichus* spp., *Philonthus carbonarius*, and *Philonthus cognatus* (see Appendix S1: Tables S4–S6 for details of all three multiplex PCR assays). The intraguild interactions, and implications on competition, have been analyzed in greater detail elsewhere (Leote et al., 2024), though only with the carabid and staphylinid diet samples, as at the time the spider gut content had not yet been molecularly analyzed; as such, it is a novel addition to the current dataset.

Following Rennstam Rubbmark et al. (2019) a diagnostic multiplex PCR was chosen over prey metabarcoding, as our regurgitate samples contain not only different amounts of DNA from several food sources, but also a lot of consumer DNA. This drastically lowers the detection of food DNA, as the primers targeting prey also bind to the taxonomically closely related consumer. Therefore, to ensure a more reliable and robust prey DNA detection, we employed the three PCR assays described above. These were also described in our previous study (Leote et al., 2024), but can also be found here in Appendix S1 for convenience.

After the multiplex PCRs, the samples were screened using capillary electrophoresis on a QIAxcel Advanced system and the ScreenGel software, with a DNA Screening Kit (2400) using a 15‐3k base pair alignment marker, following the manufacturer's recommendations. The screening profile on ScreenGel was set to the standard AM320 method, with the relative fluorescence units (RFU) detection threshold defined at 0.07, as opposed to the default 0.1, to account for the fact that the samples were extracted from gut content and thus partially digested.

### Data analysis

All data analysis was carried out using R 4.4.1 (R Core Team, 2024) and RStudio 2024.12.1+563 (Posit Team, 2025). The package *bipartite* (Blüthgen et al., 2006) was used to calculate food web network‐level specialization (H2′, an index bound between 0, for absolute generalization, and 1, for absolute specialization) and for randomized food web generation. Oksanen et al. (2022) *vegan* package was used to calculate Shannon‐Weaver's diversity of both predators and prey. The package *lme4* (Bates et al., 2015) was used for linear mixed‐effects model creation, *mgcv* (Wood et al., 2016) for generalized additive model creation, *tidyverse* (Wickham et al., 2019) for data formatting and *ggplot2* (Wickham, 2016) for graphic creation.

The diet for each predator species was averaged across all individuals of that species captured in each unique combination of sampling plot, field, and sampling session (e.g., the diet detection proportions for species A on plot 1, fertilized treatment, field 1 on sampling session 1, was the mean of all individual diet detections in that unique combination). For an example of how this was done, please see Appendix S1: Predator–prey matrix preparation example. Furthermore, self‐detections (e.g., a gut content sample from a *Poecilus* specimen testing positive for the *Poecilus* primer in PCR assay 3) were manually removed by setting all positives (value 1) to negative (value 0).

### Invertebrate diversity

Prey diversity was calculated for springtails (Collembola; Symphypleona and Arthropleona), earthworms (Annelida; Oligochaeta), gastropods (Gastropoda; Pulmonata), aphids (*Metopolophium dirhodum*, *Rhopalosiphum padi*, and *Sitobion avenae*) and the cereal leaf beetle (*Oulema melanopus*), all of which are known to be consumed by beetles and spiders (Bauer, 1982; Bryan & Wratten, 1984; Chiverton, 1987; Dennis et al., 1991; Edwards et al., 1979; Good & Giller, 2020; Harwood et al., 2004; Holopainen & Helenius, 1992; Kielty et al., 1996; Loughridge & Luff, 1983; Scheller, 1984; Seric Jelaska & Symondson, 2016; Sunderland & Vickerman, 1980); their catches can be found in Appendix S1: Table S3. Predator diversity was calculated for all *taxa* present in the wet pitfall trap sample list for 2020 and 2021, respectively (see Appendix S1: Tables S1 and S2).

### Mixed‐effects models

The null models were obtained by randomly recombining all diet data points 99,999 times, generating as many random food webs, calculating specialization for each one, then calculating Cohen's *d* standardized effect size (SES) for the difference between the random and the real food webs' specialization. This was done to provide a robust measure of how much more, or less, specialized the real food webs were than random, as opposed to simply analyzing the real specialization without a reference value.

Three main linear mixed‐effects models were carried out to test the relationship of various variables. The first model analyzed how predator diversity changed across treatments and over the sampling sessions, with the sampling field as a random factor. The second model was identical, but for prey diversity instead. The third model analyzed how specialization, as the SES described above, changed across treatments and sessions once again. As with the previous two models, the random effect was field. The stepwise model selection was done by selecting the lowest Akaike information criterion (AIC), as reported by the *lmer* function of the *lme4* package, and the significance of the variables and interaction terms was assessed through χ^2^ tests using the *anova* function of base R.

Two generalized additive models were carried out to test the effect of predator and prey diversity, respectively, on network specialization, with field and fertilization treatment as random effects. The predator and prey diversity were set as smooth terms in these models, with factor smoothing (“fs” in the *gam* function) relative to sampling session, so that all sessions had the same smoothing parameter while still allowing for different slopes. The model selection was as above, by selecting the lowest AIC, as reported by the *AIC* function of base R; following the *mgcv* package recommendations, the models were calculated using restricted maximum likelihood (REML) to correct for the smoothing parameter estimation for AIC. The significance of the diversity smooth terms was tested using the *anova.gam* function of the *mgcv* package, with *F* tests. It is worth noting that, as stated in the package manual, the resulting *p* values are approximations and should be used with caution.

Previous to testing, the heteroscedasticity of models was checked through visual inspection of quantile–quantile and residual versus predicted plots. The error structures of each model were adapted to their respective data type, with a Poisson family for counts and binomial for proportions and presence–absence data. Due to the experimental design of our study, the experimental units were the fields; thus, the sampling plots within them represent pseudoreplicates. In order to address the correlation among the plots in the same field and minimize the likelihood of falsely detecting significant differences, we followed the method in Zimmerman et al. (2021). By using mixed‐effects models with field (replicate‐level grouping variable) as a random effect, it allowed us to correct for type I error and address the pseudoreplication bias.

## RESULTS

### Food web specialization over time

**FIGURE 3:**
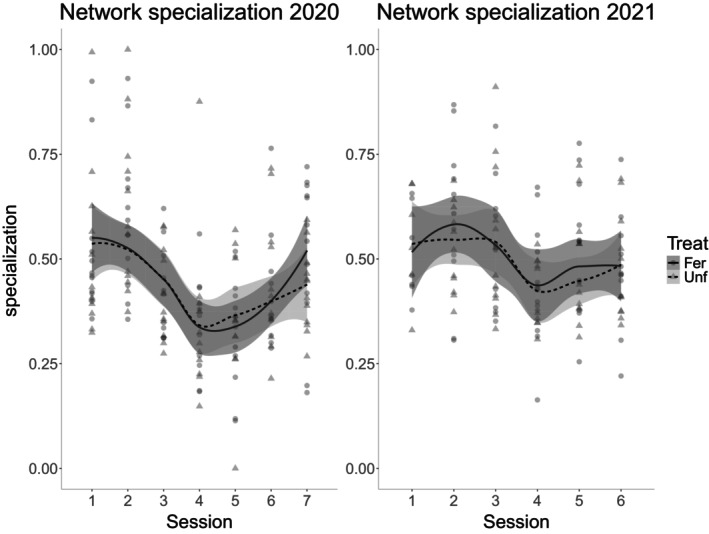
Cohen's *d* standardized effect size of the network‐level specialization (H2′) between real and random food webs over time (every 2 weeks, between 21 April and 14 July in 2020, and between 3 May and 12 July in 2021), for both fertilized (circles, full lines and darker shading) and unfertilized (triangles, dashed lines and lighter shading) treatments, in 2020 and 2021, with loess smoothing (default “loess” method in *ggplot2*) and one SE.

The positive values in the SES of the food web network‐level specialization show that it was greater than expected at random (Figure 3). As such, even though the predators analyzed were generalists, there was a degree of selection of prey. Additionally, while specialization was not significantly different in the fertilization treatment when compared to the unfertilized treatment, in both years (2020, χ^2^ = 0.305, df = 1, *N* = 165, *p* = 0.581; 2021, χ^2^ = 1.249, df = 1, *N* = 130, *p* = 0.264), it varied over time (2020, χ^2^ = 22.146, df = 6, *N* = 165, *p* = 0.001; 2021, χ^2^ = 11.715, df = 5, *N* = 130, *p* = 0.039), with a clear valley during the stem elongation phase on the 4th sampling session, meaning the predator community in general became less selective and had greater overlap in their diet. This decrease toward the middle of the season was more pronounced in 2020, but was still present in 2021, where both the initial decrease and the increase toward the end were smoother but visible (Figure 3).

### Diversity of predators and prey over time

**FIGURE 4:**
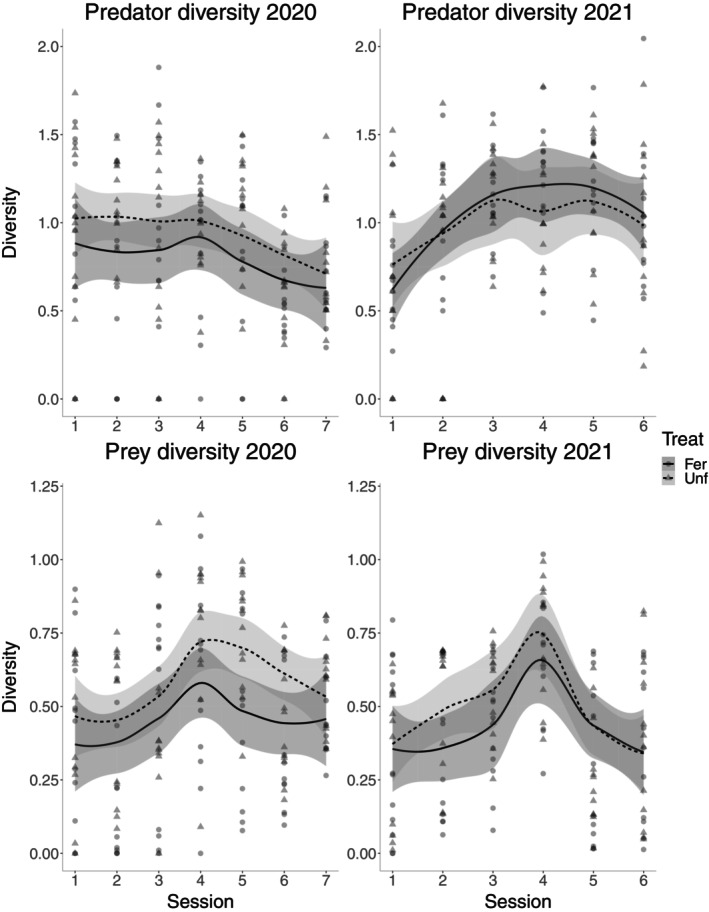
Predator and prey diversity in cereal fields over the sampling sessions (every 2 weeks, between 21 April and 14 July in 2020, and between 3 May and 12 July in 2021), for both fertilized (circles, solid lines and dark shading) and unfertilized (triangles, dashed lines and lighter shading) treatments, in 2020 and 2021, with loess smoothing (default “loess” method in *ggplot2*) and one SE.

Predator diversity was different across treatments and sessions together for 2020, being higher in the unfertilized treatment (treatment, χ^2^ = 5.658, df = 1, *N* = 168, *p* = 0.017; session, χ^2^ = 18.723, df = 6, *N* = 168, *p* = 0.005; Figure 4), but only session for 2021 (treatment, χ^2^ = 0.275, df = 1, *N* = 144, *p* = 0.602; session, χ^2^ = 25.769, df = 6, *N* = 144, *p* < 0.001; Figure 4). For prey diversity in 2020, both treatment and session had an effect, being higher in the unfertilized treatment later in the season (treatment, χ^2^ = 11.154, df = 1, *N* = 168, *p* < 0.001; session, χ^2^ = 33.807, df = 6, *N* = 168, *p* < 0.001; Figure 4), and in 2021, once again, only session had an effect (treatment, χ^2^ = 3.035, df = 1, *N* = 168, *p* = 0.082; session, χ^2^ = 53.307, df = 5, *N* = 144, *p* < 0.001; Figure 4).

### Network specialization, predator, and prey diversity

**FIGURE 5:**
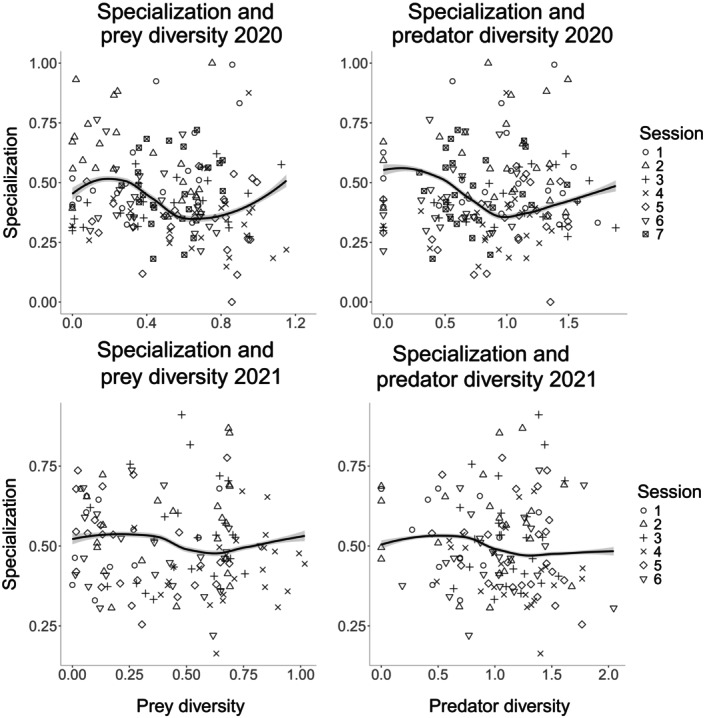
A network‐level specialization (H2′) over diversity of predator and prey in 2020 and 2021. Trend lines based on the fit and SE of the generalized additive models (GAM) with sampling session as a smoothing term to account for changes in slope, field and fertilization treatment as random effects, with loess smoothing (default “loess” method in *ggplot2*).

Network specialization was correlated to predator and prey diversity in 2020 (predator, *F* = 7.919, df = 6.700, *N* = 165, *p* < 0.001; prey, *F* = 5.213, df = 12.642, *N* = 165, *p* < 0.001; Figure 5), but no such correlation was found for prey (*F* = 1.624, df = 11.323, *N* = 130, *p* = 0.099), nor predator diversity (*F* = 2.207, df = 5.589, *N* = 130, *p* = 0.051) in 2021, albeit the latter was marginally significant (Figure 5).

## DISCUSSION

Using DNA‐based diet analysis, we were able to track the temporal dynamics within replicated invertebrate terrestrial food webs, providing unique insights into how the diet of generalist arthropod predators changes across the growing season. As these trophic data have been collected under field conditions, they reflect a portion of the natural food web dynamics, which is relevant to both fundamental and applied food web ecology. With these data, we found support for our first hypothesis that food web specialization undergoes seasonal shifts, decreasing toward mid‐season and rising again toward crop ripening. This pattern was more pronounced in 2020 but was still observable in 2021 and is likely a direct effect of the prey available to predators rather than a change in the predator community. Regarding predator diversity, it was overall lower in the fertilized treatment and decreased slightly as the season progressed in 2020, while the opposite was true for 2021, with no effect of fertilization. Prey diversity showed a peak during mid‐season for both years and was overall lower with fertilization in 2020. These patterns only partially support our second hypothesis on invertebrate diversity being highest during the middle of the season, as this only occurred for prey. Lastly, we could not find that fertilization had an effect on network specialization, but decreased predator and prey diversity, the latter only for 2020, partially disproving our third hypothesis. Additionally, we found that food web specialization was only correlated to predator and prey diversity during the first year and marginally to predator diversity in the second. Regarding the shifts of predators' behavior, there was a shift in early tillering from behavior‐constrained (high specialization) to free (low specialization) until late stem extension, then from free to constrained during late stem extension and ear forming for both years, with 2021 alone having an additional shift at the end during grain filling.

### Food web specialization and diversity

Owing to the functional link between network specialization and invertebrate diversity, we believe the specialization values at the beginning of the season were likely due to the lower prey diversity. However, prey and predator diversity were only correlated with specialization in 2020, but not in 2021. On the other hand, although only a marginal correlation was found between specialization and predator diversity in the second year, the trends for specialization and prey diversity still display a certain degree of symmetry, with the lowest values of specialization being synchronous with the highest for predator and prey diversity. Taking into account the correlation of all these factors with sampling session, it appears that phenological processes over time may be a major driver behind these changes. Based on what is known regarding the phenology of cereal fields (Zadoks et al., 1974), and invertebrate migrations into and out of them (Collins et al., 2002; Holland et al., 2004; Öberg & Ekbom, 2006; Thomas et al., 2002), it is likely that invertebrate diversity and abundance, and, by consequence, behavior, competition, and diet overlap among predators, are strongly driven by the phenology of the crops themselves. The low invertebrate diversity at the start of sampling could be attributed to the relatively sparse vegetation cover in the fields (Beaumelle et al., 2021; Rouabah et al., 2015), as it was during the tillering stage. This would be only a few weeks after the barley had been sown and the crop had not yet grown tall, so few species would have migrated into the field at that point (Öberg & Ekbom, 2006). Then, as the crop grew taller during stem extension, more species would come into the field (Collins et al., 2002; Holland et al., 2004; Thomas et al., 2002). Prey diversity increased, enabling a niche expansion of the predators, and specialization decreased toward the middle of the season, reaching its lowest value, while prey diversity reached its highest. From the 4th sampling session onward, or late stem extension and the following growth stages, diversity, particularly for prey, decreased and specialization increased for both years; coinciding with the ripening and drying of the crop, when species would be migrating out of the fields (Collins et al., 2002; Thomas et al., 2002). The link to predator diversity is less clear as, unlike for prey, the trend differed between years, decreasing throughout the season in 2020, but increasing in 2021 until the 4th sampling session; which is also reflected by the catches for each predator group, ground and rove beetles, and spiders (see Appendix S1: Figures S1 and S2).

Contrary to our expectations, manure fertilization appeared to have no effect on food web specialization in both years. However, even though we could not detect the behavioral release we expected, we have previously shown intraguild predation to be reduced and the abundances of springtails increased with manure fertilization, demonstrating an expected numerical effect of manure on both predator interference and detritivore prey (data presented in Leote et al., 2024). This shows that fertilization can have immediate effects on specific aspects of the trophic network and boost the densities of detritivores even when applied in the short term. Additionally, although we expected this short‐term application of manure to increase detritivore abundance, we also expected it would have a weak, yet positive, effect on overall invertebrate diversity; we found the opposite to be true. This is in contrast to other studies, which investigated the long‐term effect of organic fertilization on invertebrates and found that it increased general invertebrate diversity (Birkhofer et al., 2008; Kremen & Merenlender, 2018; Liang et al., 2022; Winqvist et al., 2011). The most likely explanation for these contrasting findings is that invertebrate communities take a longer time to adapt than the duration of our study, where diversity is mostly affected by species' lifecycles and movement into and out of the fields, as mentioned above. Furthermore, differences in the spreading and quantity of the manure also come into play. In our study, there was no additional manure throughout the study period, and it was spread quite finely in a thin, homogenous layer over the soil, as opposed to large pats, which provide an attractive microhabitat for invertebrates (Brévault et al., 2007) such as earthworms, springtails, ground and rove beetles (PNBL, ORRR, and MT, personal observations). Aside from detritivores, there is also the matter of omnivory, where seeds and fungi represent additional food sources. These non‐prey food sources, although beyond what we could assess with the PCR assays in our study, may contribute to reducing competition (Polis & Strong, 1996). Carbonne et al. (2020) also found that the relationship between the consumption of weed seeds and prey changes over time, depending on the prey group and its availability. Furthermore, seeds, like other alternative food sources, may increase the abundance and residence time in the fields for omnivorous species (Frank et al., 2011), which may further contribute to the CBC being provided.

### Implications for biological control management

The observed differences in food web dynamics have distinct implications regarding CBC potential by generalist arthropod predators. Fagan et al. (2002) defend that generalist predators may prevent an invasion or delay the exponential growth of pests if predator density is sufficiently high. Raymond et al. (2015) argue that, for aphids specifically, due to the damage they can cause early in the season, CBC should be strengthened during that period when pest density is lower. Following these arguments and expanding upon them based on the points made by Snyder (2019) regarding complementarity and interference, we argue the system should be managed to allow generalist predators to overlap their diets and reduce competition during the early period. To achieve this, non‐pest prey availability should be increased, and predator interference should be decreased, which we have demonstrated recently (Leote et al., 2024). That, along with low food web network‐level specialization, should increase predation pressure on pests by virtue of a greater abundance and richness of generalist predator species contributing to pest suppression.

Nonetheless, there are other treatments that can be applied as an alternative, or along with manure, to induce both greater diversity and lower specialization. These include increasing habitat structure through intercropping, cover crops, or reduced tillage to increase ground cover. This boosts invertebrate diversity and reduces predator interference, reducing behavioral constraints of predators, allowing them to be more effective biological control agents, as shown by studies (Janssen et al., 2007) and extensive reviews (Gontijo, 2019; Snyder, 2019). Most important, however, is the timing of such actions, as indicated by our results.

## CONCLUSIONS

To conclude, we have found that food web specialization fluctuated over time, almost mirroring prey, but not predators' diversity, implying that communities are highly plastic, and that redundancy was modulated within the available diversity. However, the fertilization effects were not consistent across years, and depending on the year, the predator community had different constraints at different time points. While in this study we looked mainly at the diversity of available prey and fertilization, there can also be other factors. Njue et al. (2021) found that drought affected aphid consumption by carabids and wolf spiders in barley, while Liu et al. (2018) and Beaumelle et al. (2021) found that increased vegetation structure and surrounding crops at the landscape scale play a role in biological control and predator communities. As such, organic fertilizers are only a part of the bigger picture for managing fields to increase CBC.

Such findings bring further relevance to the need to generate datasets that allow the understanding of these complex temporal dynamics of empirical food webs. This knowledge is crucial if we are to move forward, not just in the testing of models and assumptions of theoretical food webs, but also with regard to applying ecological findings to practice such as the optimization of management of pests within CBC strategies. By tracking food web changes over time and addressing the general lack of empirical studies, which deal with temporal dynamics in real‐world food webs (Isbell et al., 2018; McMeans et al., 2015; Roubinet et al., 2018), we have taken a first step toward generating the datasets needed to bridge empirical and theoretical food web science (Hooper et al., 2012; Pereira et al., 2010; Pimm et al., 2014), with direct implications for biological control.

## AUTHOR CONTRIBUTIONS

Pedro Nuno Branco Leote and Oskar Ragnar Rennstam Rubbmark carried out the field work. Pedro Nuno Branco Leote carried out the lab work. Pedro Nuno Branco Leote and Oskar Ragnar Rennstam Rubbmark conducted the data analysis. Pedro Nuno Branco Leote wrote the first draft. Oskar Ragnar Rennstam Rubbmark and Michael Traugott designed the study and procured the funding. All authors contributed to the revisions.

## CONFLICT OF INTEREST STATEMENT

The authors declare no conflicts of interest.

## Supporting information


Appendix S1.

## Data Availability

Data (Branco Leote, 2024a, 2024b, 2024c, 2024d, 2024e, 2024f) are available in Figshare at https://doi.org/10.6084/m9.figshare.26893354.v1, https://doi.org/10.6084/m9.figshare.26893405.v1, https://doi.org/10.6084/m9.figshare.26893624.v1, https://doi.org/10.6084/m9.figshare.26893630.v1, https://doi.org/10.6084/m9.figshare.26893690.v1, and https://doi.org/10.6084/m9.figshare.26893759.v1.
