# Supplementary material for: Molecular assessment of food web dynamics identifies critical periods for managing resilience in biological pest control
Source: Ecol Appl. 2025 Jul 31;35(5):e70078. doi: 10.1002/eap.70078 (PMC12313443; doi:10.1002/eap.70078)
Supplement: Supplementary file 1 — Appendix S1. [file EAP-35-e70078-s001.pdf]

Appendix S1

Molecular assessment of food web dynamics identifies critical periods for managing resilience in biological pest control

Pedro Nuno Branco Leote, Oskar Ragnar Rennstam Rubbmark, Michael Traugott

Ecological Applications

Supplementary materials

Sample collection, beetle identification and regurgitation

The beetle individuals caught in the dry pitfall traps were placed in 1.5 to 2ml tubes individually, labelled and then refrigerated in a cooling box until reaching the lab. Once there, they were taxonomically identified to the species level and made to regurgitate within their respective collection tube, after which the individual was taken from it and released outdoors. The regurgitate was buffered with TES buffer (0.1 M TRIS, 10 mM EDTA, 2% SDS; pH 8) and frozen at -80°C, until extraction. The contents of the wet pitfall traps were sieved through a fine mesh (0.5mm), rinsed with clean water, then put into a 100ml cup with 80% ethanol. Afterwards the contents were identified to the family level for most taxa, or species level in the case of beetles.

Predator species wet pitfall list

***Table S1** – Predator counts collected from wet pitfall traps in 2020.*

Species	Fertilized	Unfertilized
ARANEAE	1468	1430
STAPHYLINIDAE	305	325
Bembidion properans	89	97
Poecilus cupreus	81	96
Loricera pilicornis	27	16
Clivina fossor	22	12
Pterostichus melanarius	18	36
Poecilus versicolor	10	11
Bembidion lampros	8	4
Harpalus affinis	8	19
Nebria brevicollis	8	6
Amara aenea	6	12
Agonum muelleri	5	5
Amara bifrons	5	8
Anchomenus dorsalis	5	4
Bembidion tetracolums	5	16
Bembidion quadrimaculatum	4	8
Harpalus rufipes	4	9
Amara similata	2	11
Anisodactylus binotatus	2	2
Agonum sexpunctatum	1	1
Amara familiaris	1	0
Harpalus distinguendus	1	1
Harpalus griseus	1	0
Harpalus latus	1	0
Amara montivaga	0	1
Anisodactylus signatus	0	1
Carabus granulatus	0	2

***Table S2** – Predator counts collected from wet pitfall traps in 2021.*

Species	Fertilized	Unfertilized
ARANEAE	703	708
Bembidion properans	178	88
STAPHYLINIDAE	169	199
Amara aenea	67	42
Amara similata	66	159
Poecilus cupreus	57	84
Pterostichus melanarius	30	70
Harpalus affinis	14	10
Clivina fossor	12	13
Nebria brevicollis	12	10
Poecilus versicolor	10	10
Bembidion tetracolums	9	0
Bembidion quadrimaculatum	6	2
Loricera pilicornis	6	6
Anchomenus dorsalis	4	3
Agonum sexpunctatum	3	5
Anisodactylus binotatus	3	0
Harpalus rufipes	3	4
Cylindera germanica	2	3
Amara familiaris	1	0
Bembidion lampros	1	2
Calathus melanocephalus	1	0
Harpalus caliginosus	1	0
Harpalus distinguendus	1	0
Calathus fuscipes	0	1

Table S3 *– Prey counts collected from wet pitfall traps in 2020 and 2021.*

	2020		2021	
Species	Fertilized	Unfertilized	Fertilized	Unfertilized
ACARI	2529	2276	363	423
APHIDIDAE	772	996	87	54
COLLEMBOLA ARTHROPLEONA	28448	10288	25753	14601
COLLEMBOLA SYMPHYPLEONA	3664	6641	3148	3785
GASTROPODA	7	5	0	0
ISOPODA	33	13	0	2
LUMBRICIDAE	2	14	0	0
MYRIAPODA	36	45	31	38
Oulema melanopus	11	7	9	7
RYNCHOTA	296	285	129	166
THYSANOPTERA	3	6	5	6

Multiplex PCR assays

- Multiplex PCR 1

The first multiplex PCR mix was comprised of 1µl PCR-grade water, 5µl KAPA2G FAST HotStart® DNA Polymerase (Roche), 1µl of the primer mix (supplementary table 1), 0.5µl of 10mg/ml BSA and 2.5µl of template DNA, for a total volume of 10µl. The thermocycling was done in a Mastercycler ® Nexus Gradient (Eppendorf, Hamburg, Germany), with the following conditions: 15 min at 95 °C, 35 cycles of 30 s at 95 °C, 90 s at 62.5 °C and 30 s at 72 °C, and 10 min at 72 °C.

Table S4 *– Primer list for multiplex PCR 1. + from Staudacher* et al. *(2016), * from Ye* et al. *(2017), ** Kuusk & Agusti (2008), *** from Rennstam Rubbmark* et al. *(2019).*

**Target**	**Primer code**	**Gene**	**Product (bp)**	**Final conc. in PCR (S/AS)(µM)**	**Primer sequence (5'-3')**
Earthworms	S408+	18S	85	0.8	CGTAGTTGGATCTCGGGTCGT
	A413+				ATARGGGTCGGAGCTTTGTG
Metopolophium dirhodum	S436*	COI	105	0.4	CCTTTATCAAATAACATTGCACATAAC
	A440*				AATAAAGTTAATTGCTCCTAAAATTGAG
Rhopalosiphum padi	S440*	COI	136	0.3	TAATAATATAAAATTAAACCAAATTCCATTA
	A442*				TGATGTATTTAAATTACGATCAGTAAGAAG
Acyrthosiphon pisum	S492***	COI	210	0.08	GTCCTGATATATCATTTCCTCGC
	A496***				AAATTGATGAAATTCCTGCTAGG
Springtails	Col3F**	18S	231	0.2	GGACGATYTTRTTRGTTCG
	A415+				GAATTTCACCTCTAACGTCGCAG
Oulema melanopus	Om-S2-KS-S185***	COI	248	0.2	TTGACTTCTCCCACCTTCAA
	Om-A-KS-A184***				CAAACAGAGGCATTCGATCT
Sitobion avenae	S433*	COI	304	0.1	TCATCACTTAGAATTCTTATTCGTCTT
	A438*				AAGGTGGRTAAATAGTTCATCCTGTA

- Multiplex PCR 2

The second PCR mix was comprised of 0.7µl PCR-grade water, 5µl of QIAGEN Multiplex PCR Master Mix (Qiagen), 1µl of the primer mix (supplementary table 2), 1µl Q-solution (Qiagen), 0.5µl of 10mg/ml BSA, 0.3µl of 30mM TMAC (Sigma-Aldrich) and 1.5µl of template DNA, for a total volume of 10µl (supplementary table 4). The thermocycling conditions were as follows: 15 min at 95 °C, 35 cycles of 30 s at 94 °C, 3 min at 62 °C and 90 s at 72 °C, and 10 min at 72 °C (supplementary table 6).

Table S5 *– Primer list for multiplex PCR 2. + primers from Sint* et al. *(2014), * primers from Staudacher* et al. *(2016).*

**Target**	**Primer codes**	**Gene**	**Product (bp)**	**Final conc. in PCR (S/AS)(µM)**	**Primer sequence (5'-3')**
Hoverflies	S269+	18S	86	0.9	ATTAGGCTAAAACCAAGCGATTT
	A270+				TCGGTACAAGACCATACGATCG
Lacewings	S417*	18S	390	0.4	CTGTGTCCTACACTGTTGGTTCAAT
	A420*				AATGCCCCCATCTGTCCG
Spiders	S407*	18S	~258	0.3	AATAACRATACGGGACTCTTTYGAGA
	A408*				CGAGACAACCGGTRAAGATCAT
Beetles/thrips	S405*	18S	~208	0.05	ACAGAGCTCYGACCGGAGAC
	A406*				TTACAACCATGGTAGGCGCAG
Ladybeetles	S415*	18S	116	1.2	CCCAAHTKDCCCCGC
	A418*				GCATAAAATATTCYGGCAAAATTTC

- Multiplex PCR 3

The first multiplex PCR mix was comprised of 1.7µl of PCR-grade water, 5µl Type-it Multiplex PCR Master Mix (Qiagen), 1µl of the primer mix (supplementary table 3), 0.5µl of 10mg/ml BSA, 0.3µl of 30mM TMAC (Sigma-Aldrich) and 1.5µl of template DNA, for a total volume of 10µl. The thermocycling conditions were as follows: 5 min at 95 °C, 35 cycles of 30 s at 95 °C, 3 min at 63 °C and 90 s at 72 °C, and 10 min at 72 °C.

Table S6 *– Primer list for multiplex PCR 3. * Primers developed for this study, all others from Staudacher* et al. *(2016).*

**Target**	**Primer codes**	**Gene**	**Product (bp)**	**Final conc. in PCR (S/AS)(µM)**	**Primer sequence (5'-3')**
Poecilus	S475	COI	112	0.15/0.15	GTGCATGATCAGGAATAGTRGGT
	A486				GCAGTAACAATAACATTATAAATTTGATCG
Pterostichus	S467	18S	166	0.4/0.2	TGATCTCGAAACGGGTCTTTTACT
	A467.1				CCTGTTYCATTATTCCMTGCACTA
Bembidion	S468	18S	~152	0.1/0.1	TGTTTAACTGGCACGTCTCGC
	A470				GCACCGCGACAGGATTATTG
Harpalus	S473	COI	349	0.8/0.8	GCAGGAATAGTAGGAACTTCATTAAGC
	A475				AAGCTCCTCTATGWGCRATTCC
Philonthus cognatus	S927*	COI	216	0.4/0.4	TAGCTGGGATTTCCTCAATTCTC
	A927*				CTGTTGGGTCAAAGAATGTTGTG
Philonthus carbonarius	S928*	COI	245	0.2/0.2	AGAACTAGGAAACCCCGGTACA
	A928*				CGCTTTCAACTATTCTTCTCATTAAA

Multiplex PCR assays references

Kuusk, A. K., & Agusti, N. (2008). Group-specific primers for DNA-based detection of springtails (Hexapoda: Collembola) within predator gut contents. Molecular Ecology Resources, 8(3), 678–681. https://doi.org/10.1111/j.1471-8286.2007.02046.x

Rennstam Rubbmark, O. R., Sint, D., Cupic, S., & Traugott, M. (2019). When to use next generation sequencing or diagnostic PCR in diet analyses. Molecular Ecology Resources, 19(2), 388. https://doi.org/10.1111/1755-0998.12974

Sint, D., Niederklapfer, B., Kaufmann, R., & Traugott, M. (2014). Group-specific multiplex PCR detection systems for the identification of flying insect prey. PloS one, 9(12), e115501. https://doi.org/10.1371/journal.pone.0115501

Staudacher, K., Jonsson, M., & Traugott, M. (2016). Diagnostic PCR assays to unravel food web interactions in cereal crops with focus on biological control of aphids. Journal of pest science, 89, 281-293. https://doi.org/10.1007/s10340-015-0685-8

Ye, Z., Vollhardt, I. M., Girtler, S., Wallinger, C., Tomanovic, Z., & Traugott, M. (2017). An effective molecular approach for assessing cereal aphid-parasitoid-endosymbiont networks. Scientific Reports, 7(1), 3138. https://doi.org/10.1038/s41598-017-02226-w

Predator species regurgitates list

***Table S7** – Ground beetle (Carabidae) and rove beetle (Staphylinidae) regurgitate samples collected in 2020.*

Species	Fertilized	Unfertilized
Poecilus cupreus	436	380
Bembidion properans	363	285
Philonthus cognatus	353	382
Philonthus carbonarius	104	74
Loricera pilicornis	56	54
Bembidion lampros	52	59
Pterostichus melanarius	51	72
Amara aenea	46	71
Bembidion tetracolum	26	37
Agonum muelleri	21	35
Bembidion quadrimaculatum	20	12
Clivina fossor	20	27
Poecilus versicolor	19	22
Anchomenus dorsalis	17	35
Harpalus affinis	16	33
Harpalus rufipes	15	13
Nebria brevicollis	8	12
Amara ingenua	5	6
Anisodactylus binotatus	5	6
Amara similata	4	18
Amara sp.	4	7
Calathus fuscipes	3	2
Agonum sexpunctatum	2	2
Carabus granulatus	2	2
Harpalus distinguendus	2	2
Amara familiaris	1	5
Amara plebeja	1	0
Bembidion sp.	1	0
Cylindera germanica	1	0
Harpalus sp.	1	0
Amara lucida	0	1
Amara lunicollis	0	1
Anisodactylus signatus	0	3
Bembidion metallina	0	1
Bembidion pilicornis	0	1
Calathus melanocephalus	0	1

Table S8 *– Ground beetle (Carabidae) and rove beetle (Staphylinidae) regurgitate samples collected in 2021.*

Species	Fertilized	Unfertilized
Philonthus cognatus	369	355
Poecilus cupreus	255	281
Bembidion properans	229	155
Pterostichus melanarius	197	217
Amara aenea	87	62
Philonthus carbonarius	69	70
Amara similata	53	91
Poecilus versicolor	35	33
Nebria brevicollis	25	27
Anisodactilus binotatus	16	13
Bembidion lampros	16	11
Harpalus affinis	15	9
Loricera pillicornis	15	16
Agonum muelleri	10	4
Clivina fossor	10	18
Tachyporus ruficollis	10	17
Harpalus rufipes	9	9
Agonum sexpunctatum	7	1
Bembidion quadrimaculatum	7	0
Anchomenus dorsalis	5	12
Bembidion tetracolum	4	1
Carabus granulatus	4	1
Amara familiaris	3	0
Cylindera germanica	3	1
Anisodactilus signatus	1	3
Calathus fuscipes	1	3
Harpalus distinguendus	1	0
Calathus melanocephalus	0	1
Tachyporus rufipes	0	1
